# Navigating Complexity: Lessons Learned from Co-Designing a Care Transition Intervention for People with Stroke

**DOI:** 10.5334/ijic.8943

**Published:** 2025-04-24

**Authors:** Sebastian Lindblom, Charlotte Ytterberg, Ann Charlotte Laska, Malin Tistad, Marie Elf, Lena von Koch, Maria Flink

**Affiliations:** 1 Department of Neurobiology, Care Sciences and Society, Karolinska Institutet, 14183 Stockholm, Sweden; 2 Theme of Women’s Health and Allied Health Professionals, Karolinska University Hospital, 14186 Stockholm, Sweden; 3 Department of Clinical Sciences Danderyd Hospital, Karolinska Institutet, 18288 Stockholm, Sweden; 4 School of Health and Welfare, Dalarna University, 79131 Falun, Sweden; 5 Theme of Heart & Vascular and Neuro, Karolinska University Hospital, 14186 Stockholm, Sweden; 6 Research and Development Unit for Elderly Persons (FOU nu), Region Stockholm, 177 31 Järfälla, Sweden

**Keywords:** adaptive systems, co-design, collaboration, complexity, emergence, integrated care, involvement

## Abstract

**Introduction:**

Participatory, emergent, and reflective approaches are needed in research on person-centred integrated care. This paper describes and explores the process of developing a complex care transition intervention for stroke survivors, along with the lessons learned.

**Description:**

This study concerns the Missing Link project, which aimed to develop and evaluate a care transition intervention from hospital to home. The care transition was developed according to the Medical Research Council (MRC) Framework and included studies on context, co-design workshops, and prototype development.

**Discussion:**

The development process faced challenges relating to understanding the emergence within the studied context and the complex adaptive systems. We failed to have a continuous and sustained involvement of healthcare professionals, patients, and significant others during the different phases of the project. Hence, a lack of shared understanding is conceivable as the emergence might have been interpreted and understood differently by the actors.

**Conclusion:**

Challenges in achieving shared understanding throughout the project underline the importance of investing in relationship building, meaningful interaction, and continuous feedback loops. While the MRC framework provides guidance for developing complex interventions, the phased approach may only partially capture the emergence and self-organisation within complex adaptive systems.

## Introduction

During recent years, calls have been made for research in person-centred integrated care that includes a wider “epistemic fluency” with more participatory, emergent and reflective approaches [1]. Such approaches are better suited to inform our understanding of the complex and dynamic interactions within person-centred integrated care [2]. Participatory approaches may also facilitate the development of novel interventions, such as care transitions, which respond to real-world contexts.

Care transitions from hospital to home for people with stroke occurs within complex and adaptive systems [3] and includes several challenges: coordination of care, information focused on supporting patient understanding, and preparedness for home-coming [4,5,6]. For patients and their significant others, uncoordinated care transitions are challenging, especially when they lack knowledge on navigating the healthcare system [7]. Information is the largest unmet need for people with stroke [8]; and several sequels, such as cognitive impairments, post-stroke fatigue and depression [9] make understanding information particularly difficult. For older people, i.e., the majority of stroke survivors, the lack of knowledge and information post-discharge is a major concern: up to 62% lack knowledge of their new medications [10] and > 50% cannot remember the plan for follow-up [11]. People with stroke have thereto reported feeling unprepared for the care transition to home [12,13] and abandoned by healthcare after discharge [6]. These multifaced challenges call for complex, multi-component interventions to improve the care transition from hospital to home for people with stroke [14]. Designing these interventions is in itself a complex process that involves numerous agents with different perspectives and agendas, all of whom have the freedom to act, leading to unpredictable behaviours and actions.

Since the introduction of the first Medical Research Council (MRC) framework in 2000 [15], there has been an increasing awareness that complex problems require complex interventions, especially when creating person-centred integrated care models. Developing such interventions is a non-linear process of identifying key uncertainties, involving stakeholders, considering context, and iterative refinement [16]. The MRC framework recommends the use of co-design with patients, family members, and professionals in the development of complex interventions [16,17,18] to increase the likelihood of meeting the needs of the users and fitting the contextual conditions [19]. To avoid research waste, the MRC framework highlights that thorough descriptions of intervention development are needed to guide the development of future interventions that aim to integrate existing evidence [16]. There is also a need to further understand the “black box” of complexities that the development of person-centred integrated care initiatives within complex adaptive systems presents [20,21].

In our Missing link project [14], we used multiple methods and strategies of participatory approaches in developing an intervention to support the transition to home for people with stroke in Swedish health services. This paper describes and explores the process and lessons learned while developing a complex care transition intervention for people with stroke.

## Study context

This study within the Missing Link project was conducted in Stockholm, Sweden. Missing Link aimed to explore current care transitions and develop and evaluate a new care transition intervention from stroke units to rehabilitation at home, whereof this study concerns an exploration of the current care transitions and the development of a new intervention.

In Sweden, hospital care and rehabilitation are publicly funded [22]. Provision of care is prioritised based on the principles of human dignity, needs and solidarity, and cost-effectiveness. Most patients with acute stroke are treated in stoke units [23]. After the acute hospital stay, patients are discharged to care and rehabilitation either as in-patients at rehabilitation or geriatric units or to their homes with visits by a neuro-rehabilitation team from primary care.

The Missing Link project concerns the care trajectory of patients who are discharged to home either directly from the acute stroke unit or from a geriatric unit; it has previously been described in detail [24]. At discharge to home, a referral is sent to the neuro-rehabilitation team consisting of a physiotherapist, occupational therapist, speech and language therapist, and social worker. The team is obligated to contact the patient within 48 hours after the discharge. A referral with a request for follow-up is also sent from the hospital to the primary care physician. However, the neuro-rehabilitation team and the primary care physician are not part of the same organisational units. In addition, some hospitals, but not all, offer an out-patient follow-up after the acute care.

## Study methodology and framework

The Missing Link project was developed based on the MRC framework for developing and evaluating complex interventions [16], including the following phases: the development phase, which comprises identifying the evidence base, understanding the context, and co-designing the intervention; the feasibility and piloting phase, which comprises testing procedures, estimating recruitment and retention, and refining the intervention; the evaluation phase which comprises evaluating and assessing effectiveness, understanding mechanisms of action, and considering implementation processes; and the implementation phase considering how to scale up, assessing long-term impacts, and how to integrate the intervention into practice.

This study focuses solely on the development phase, which comprises three phases: pre-studies to explore and understand the context, co-design workshops, and prototype development. In the following section, we elaborate on the development of the complex care transition intervention for people with stroke by describing each step and reflecting on the strengths, weaknesses, and lessons learned. The following reflections have been developed through iterative discussion between the researchers in the Missing Link project i.e., the authors of this paper.

## Description of the development of the care transition intervention

### Pre-study

Three pre-studies were performed to explore facilitators and barriers in the current care transition between hospital and rehabilitation at home regarding both process and outcomes: a cross-sectional [25], prospective observational [24] and qualitative [5] study. The studies were performed in parallel between April 2016 and February 2018. Patients with stroke, n=206, who were to be discharged from acute stroke units with referral to a neurorehabilitation team in primary care, were recruited along with their significant others, n=89. Data collection at baseline and at one week, three- and 12-months post-discharge comprised patient characteristics, disease-related data and functioning. Register-based data on the use of healthcare services were collected up to 12 months post-discharge. Data from significant others were collected on informal care supplied, caregiver burden, and life satisfaction at three- and 12-months post-discharge. The qualitative study [5] explored the perspectives of the care transition from stroke survivors, healthcare professionals, and significant others using focus groups, individual and dyad interviews with a total of 71 participants.

The findings of the pre-studies showed that stroke survivors and significant others did not feel prepared for home-coming and the responsibility to self-manage their condition. Communicating information on medications before hospital discharge was identified as a particular need for attention to improve the overall perceived quality of the care transition. Stroke survivors, significant others and healthcare professionals experienced a need for improved dialogue throughout the care transition: between patients and professionals; between professionals in the same organisation; and between professionals in different organisations. There were large variations in the use of healthcare services, and no single pattern of amount of usage was identified. Instead, the use of healthcare services seemed to mirror the functioning of stroke survivors.

#### Reflections

A strength of the pre-study phase was the use of both quantitative and qualitative methods, allowing for an understanding of the current care transition from different perspectives. This gave us insights that the intervention to be developed should focus on preparedness for home-coming and shared understanding between all involved stakeholders. The care transition involved multiple stakeholders, i.e., patients, significant others and healthcare professionals of different professions and organisations, who had to coordinate their perspectives during a short period of time. We therefore concluded that a co-design process with all these stakeholders was necessary to enable a development of a new care transition that met the needs of all involved. However, we should have conducted the qualitative study before the quantitative observational study and collected more in-depth data on the discharge procedures and contextual factors in the studied clinical setting. This would have offered a more profound insights on the daily procedures at the hospitals, including the problems and challenges experienced by the professionals in their daily work. This may have steered the data collection in the observational study towards outcome measures of patient involvement in stroke care and home-rehabilitation, self-management, and perceived person-centeredness. As now, we planned the observational study and its measurements based on the previous literature on Early Supported Discharge [26], in which the outcomes are mainly focused on patient and caregiver functioning post-discharge and resource use of healthcare. If we had spent more time in the clinical settings, we could also have strengthened the opportunity to understand the professionals’ perspectives of changes needed in the care transition. Equally important, we could have established relationships with the healthcare professionals that we continued working with in the following phases of the project. This might have better established a mutual understanding of the problems needed to be solved.

### Co-design workshops

Based on the knowledge generated from our pre-studies we conducted a co-design process with stroke survivors, significant others, and healthcare professionals to design a new care transition process between hospital and continued home-rehabilitation. The co-design conducted from November 2019 to January 2020 used design thinking [27] and followed the five steps of the double-diamond model [28]: 1) *The empathy phase*, which sought to create a mutual understanding, empathy, and trust between the users through using patient narratives, interviews and exploring and mapping the patient journey; 2) *The problem framing*, with the intention to identify and synthesise the needs, and define the aims and challenges through methods such as in-depth interviews, exploration of needs, and “how might we questions”; 3) *The ideate phase*, which focused on generating ideas, and defining, analysing, and prioritising ideas through brainstorming sessions, SWOT-analysis, idea cards, and service blueprints; 4) *The prototype phase* (described below), with the aim to reconceptualise needs, adjust ideas, concretise solutions, and create prototypes through creation of storyboards, creative prototyping, and feedback iteration; and 5) *The test phase* (not part of this paper), where the solutions and prototypes were tested through role-play, focus groups, pitching and evaluation sessions. The workshops were led by an external facilitator trained in design thinking, but without experience of facilitating patient groups, and held at a centre for health innovation external to the healthcare organisations.

Three stroke survivors and one significant other were recruited to participate in the workshops through the two patient organisations Swedish Stroke Association and Neuro Sweden. Nine healthcare professionals were recruited from in-patient units at one hospital (one of the hospitals in the pre-studies) and two corresponding neurorehabilitation teams in primary care consisting of a physician, physiotherapists, a registered nurse, speech and language therapists and occupational therapists.

The participants were divided into three groups, the same during all workshops, and three researchers participated as observers of one group each. The co-design process aimed to create solutions that meet the needs of stroke survivors and their significant others and develop a person-centred and coordinated care transition from hospital to home. The co-design process was initiated with a presentation of the main findings from the pre-studies through an oral presentation and written patient narratives. No pre-decided direction on the problem-framing and idea generation was provided. Three areas of needs with related solutions for improvement were generated, one from each group:

1) A need for coordination between the hospital care and the neurorehabilitation teams. To address this need, a bridging e-meeting before discharge, including scheduling the follow-up visit, was suggested; 2) a need for shared understanding between health professionals and patients concerning patient needs. To address this need, structured and timely verbal and written information; increased involvement of patients and significant others; and communication adapted to the situation were suggested; and 3) a need for patients to feel safe and prepared for home-coming at discharge. To address this need, an easily accessible and informative person-centred document to provide patients with an overview of the different steps of the care transition process after stroke was suggested.

#### Reflections

The strength of the co-design workshops was the participation of all stakeholders involved in the care transition. The use of three smaller groups facilitated the discussions within each group, allowing all stakeholders to get to know each other and therefore more easily make their voices heard. Another strength was that the workshops took place at a setting outside the healthcare organisations with a neutral facilitator, i.e., no organisational perspective was given priority over the others.

However, neither the facilitator nor the researchers had practical experience of co-design with such a mixed group of patients, significant others, and healthcare professionals. Thereto, we as researchers deliberately took a passive role as observers to be able to collect data on the co-design process for a paper on the manifestation of participation within co-design [29]. We believe that the combination of a lack of experience and us assuming the role of passive observers hindered the facilitation of stakeholders’ interaction in the co-design process. In retrospect, the importance of facilitators’ tailoring the activities within the process to stakeholders with a varied ability to participate in workshop discussions, and the need for the continuous active involvement of facilitators in all groups, has become evident to us.

One of the groups concluded towards the end of the co-design process that they needed more information on patients’ experiences of the care transitions. In hindsight, we believe that the co-design phase could have been improved with a prolonged and deepened empathy phase to deepen the possibility for mutual understanding, empathy and trust between users, such as involving the healthcare professionals in conducting the pre-study interviews. This would have allowed the healthcare professionals to familiarise themselves with and become more aware of the needs to improve the care transitions from the patient perspective. Further, a larger representation of stroke survivors and significant others could have increased the understanding of patient experiences. As this would have meant larger co-design groups, possibly hampering the collaboration within the group, we could have organised the co-design differently, for example, with both mixed groups and with separate patient, significant others, and professional groups.

### Prototype development

The suggested solutions for improvement from the co-design were further developed into more mature prototypes in meetings within the research group, in meetings with managers, in meetings with healthcare professionals from the co-design phase, and new representatives from the acute stroke unit, geriatric unit and neurorehabilitation teams. This iterative development phase ranged from February 2020 to November 2021. In total, five meetings with managers in organisationally specific groups and 22 meetings with healthcare professionals both in cross-organisational and organisational specific groups were held to further develop the prototypes. In addition, 16 meetings with healthcare professionals in cross-organisational and organisational specific groups, and two meetings with managers, were conducted to refine and implement the prototypes. All meetings except one were conducted using Zoom due to the pandemic.

The prototype development phase resulted in a care transition model with several interacting components [30]: 1) Information on the neurorehabilitation team provided to patients at the acute stroke unit or geriatric unit prior to discharge using information videos and a pamphlet; 2) a dialogue between healthcare professionals and the patient on “what matters to me” regarding home-coming and continued rehabilitation; 3) a rehabilitation plan established based on the outcomes of the “what matters to me” dialogue; 4) a bridging e-meeting before hospital discharge between the patient and the neurorehabilitation team where the appointment for the first home visit is scheduled; 5) a template for a patient-friendly discharge summary to be used during the discharge encounter; 6) “Teach Back” [31] used in all communication between healthcare professionals and patients to ensure a shared understanding; and 7) instant, direct messages within the electronic medical record system between the hospital and neurorehabilitation team.

#### Reflections

A strength of the prototype development phase was that it continued directly after the co-design workshops, allowing for a concretisation of the suggested solutions in the clinical settings. Thereto, the longitudinal iterative development with both professionals and managers enabled us to anchor the project in the clinical settings.

A challenge in the prototype development was the changed staffing situation. The researcher most involved in the co-design process and in contact with the co-design participants could not be as much involved as previously. This caused a gap in the intrinsic knowledge of the project as well as on the established relations with the participants. Further, the healthcare professionals had a high workload because of the pandemic, and some professionals participated on demand by their managers rather than based on a personal motivation. The researchers thus had sole responsibility for refining the prototypes and presenting them to the healthcare professionals to get feedback. We reflect that important perspectives in the development of prototypes may therefore have been missed, leading to prototypes that were not fully compatible with the clinical setting.

A major lack in the prototype development was the absence of participating stroke survivors and significant others in the prototype development phase. We deliberately chose to not include the stroke survivors and the significant other from the co-design to open for new perspectives. We approached patient organisations but were only able to recruit one stroke survivor with no recent experiences of care transitions.

## Discussion

The overall aim of the Missing Link project was to develop a person-centred care transition intervention for people with stroke. The present study aimed to describe the development of this complex intervention, and the lessons learned from the process.

When following the MRC framework for developing a complex intervention with the intention of using a participatory, emergent and reflective approach [16], we ran into some challenges relating to understanding the emergence within the studied context. Reasons for this could be the well-known disconnect between academics and practitioners [32] and the lack of embeddedness in the real-world context that our phased approach generated. Despite our knowledge on the importance of stakeholder involvement throughout the development process [18], we failed to have a continuous and sustained involvement of healthcare professionals, patients and significant others during the different phases of the project. As a result, the needs and priorities generated in the different phases might have been interpreted and understood differently by the actors, i.e., a lack of shared understanding. Issues relating to the fact that actors in different sectors vary in their perspectives are well-known, sometimes referred to as institutional logics. Institutional logics relates to organisations having socially constructed patterns of, for example, ideas, values, and beliefs that shape how actors within the organisations behave and perceive their activities and experiences [33]. It has been suggested that interpersonal relationships between actors can overcome competing institutional logics [34]. The limited opportunities for the stakeholders in our study to create conditions that allowed for inter-personal relationships and understanding of each other’s different logics affected the possibility for involvement, collective action and collaboration, something that has been proposed as an essential aspect in stakeholder involvement within complex interventions [35]. We should have put more effort into enabling arenas for interaction and connectivity to support the development of relationships between stakeholders as these factors have also been shown to influence partnership and collaboration in healthcare improvement and implementation [36,37]. Involvement of stakeholders in the development of complex interventions has been highlighted as crucial to get a common understanding of the problem and the needed solutions [16,18].

We initiated the development of the new care transition based on needs identified in scientific literature, without sufficient knowledge on the daily procedures in the clinical settings and stakeholders’ experiences. A discussion with stakeholders in the beginning of the project on their perspective of the problems with current care transitions, and what they wished to contribute with and achieve with participating in the project [38], could have facilitated a shared understanding of the project goals. Spending more time in the clinical setting for a better understanding of the stakeholders’ needs could have been time well spent, as time has been identified as prerequisites for genuine partnership between researchers and stakeholders when conducting co-design processes [39]. Another way of ensuring that stakeholders and researchers agree upon the focus of the research could have been to involve healthcare professionals, stroke survivors and significant others in conducting interviews of stakeholders as part of the qualitative pre-study [19]. The lack of shared understanding of the project goals in our study are in line with Lee et al., who found that it is challenging for stakeholders to be involved in the development of projects when the problems to solve are not well defined [40]. Hence, deliberate efforts from project start to create a shared understanding of the problem needed to be solved is a prerequisite for the development of complex interventions within and with clinical settings. Another challenge in the development of complex healthcare interventions is that the process is time-consuming and stretches over a long period of time [41]. Involvement of healthcare professionals is, in general, demanding due to lack of staff and high staff turnover in healthcare [42]. In addition, as our project was conducted during the pandemic, this was extremely challenging. To handle the risk of professionals dropping out of the project we tried to balance how much we could involve them in the development without burdening them too much. However, in doing so we may have gone too far in protecting their time [38] and thereby did not follow the principles of patient and public involvement as we partly took back the control and power that we wanted to share with stakeholders [43]. Further, as the development stretched over a long period of time, it was challenging for staff to maintain motivation, interest, and engagement. We did not articulate an overview of the different steps of the development, which may have caused a lack of clarity of what the staff’s involvement would consist of and lead to [38,39]. Without feedback from researchers on how staff involvement contributes to the development, staff may lack motivation to continued involvement [41]. Quick decision-making processes have been suggested as a way of working to handle time-pressure when involving healthcare professionals [39]. When conducting long-term development projects, researchers need to have an outlined, clear strategy on how to maintain stakeholders’ motivation and engagement, without burdening them. Such a strategy should include a shared understanding of the extent and type of involvement in the project’s different phases, continuous feedback loops between researchers and stakeholders, and plans for how to handle stakeholder turnover to ensure continuity.

Another challenge with projects being time-consuming and stretched over a long period of time [41] is to maintain a representation of all stakeholders’ perspectives. The challenges with recruiting and maintaining attendance for “hard to reach” groups, such as people with disabilities, are well known [43]. Maintaining patient and significant other perspectives in projects on care transition for patients with stroke may be particularly challenging. Usually, a stroke is a life changing one-time event. For patients with recent care transitions after stroke, it may be too challenging to be actively involved in developing processes, as they must manage the changed life situation. On the other hand, for people with long past experiences of the care transition after stroke, the event may be difficult to recall in detail. We therefore argue that it is crucial in the recruitment process to discuss beforehand with potential participants what the involvement entails so they can make an informed decision about their participation. In addition, in the case of care transitions after stroke, it may be necessary to include more individuals as the one-time experience of care transitions can be experienced very differently and therefore needs to be explored from several individual perspectives to ensure a diversity of background, perspectives, and thoughts [38]. Equally important, once the recruitment is completed, the researchers need to be adaptive to the participants’ diverse prerequisites and the emergence of the co-design process.

The strength of using the MRC framework for developing a complex intervention has been the possibility to incorporate both quantitative, qualitative, and participatory methods to understand the current care transition from different perspectives. Further our phased approach made it possible to build each phase on knowledge generated from the previous one. However, this approach also meant simplifying the complex adaptive system as it divided the development process into several singular entities. Instead, an embedded researcher approach might have led to a more in-depth understanding of the emergence within the studied context and facilitated a shared understanding between the actors [44].

### Lessons learned

- Do not underestimate the time needed to understand the context and the needs of the involved stakeholders.
- It is crucial to enable arenas for interaction and connectivity to support relationship building and involvement, especially in relation to developing care transitions.
- It is of critical importance to consider and plan for how feedback loops can be used to ensure a shared understanding throughout the development of complex interventions.
- A coherent development process is important to avoid stakeholder fatigue.

## Conclusion

Developing a person-centred integrated care intervention for people with stroke in complex adaptive systems presents both challenges and opportunities. Despite following the MRC framework for developing a complex intervention with the intention of using a participatory, emergent, and reflective approach, the development process faced several challenges concerning the emergence of a shared understanding throughout the different phases of the project. It is crucial to invest time and resources in understanding the context and needs of all stakeholders involved. Involving diverse stakeholders necessitates meaningful interaction and connectivity among them to facilitate relationship building, collaboration, and shared understanding of project goals and processes. Moreover, continuous feedback loops need to be incorporated throughout the process to ensure a shared understanding and adaptation to the real-world context. While complex intervention development requires time, excessively long processes can lead to stakeholder fatigue. Therefore, consideration needs to be taken to avoid prolonged timeframes. Although the MRC framework provides structure and guidance for developing complex interventions, the phased approach may only partially capture the emergency and self-organisation within complex adaptive systems. In summary, successful complex interventions demand stakeholder collaboration, adaptive strategies, and a nuanced understanding of emergent systems. Future development of person-centred integrated care initiatives within complex adaptive systems should use existing frameworks for complex interventions but must continuously refine their approaches with a focus on incorporating methods suitable for understanding the emergence that the real world involves.
